# Bilaterally positive head-impulse tests can differentiate AICA infarction from labyrinthitis

**DOI:** 10.3389/fneur.2024.1448989

**Published:** 2024-08-29

**Authors:** Sung-Hwan Kim, Hanseob Kim, Sun-Uk Lee, Euyhyun Park, Bang-Hoon Cho, Kyung-Hee Cho, Gerard J. Kim, Sungwook Yu, Ji-Soo Kim

**Affiliations:** ^1^Department of Neurology, Korea University Medical Center, Seoul, Republic of Korea; ^2^Department of Computer Science and Engineering, Korea University, Seoul, Republic of Korea; ^3^Neurotology and Neuro-ophthalmology Laboratory, Korea University Medical Center, Seoul, Republic of Korea; ^4^Department of Otorhinolaryngology-Head and Neck Surgery, Korea University College of Medicine, Seoul, Republic of Korea; ^5^Dizziness Center, Clinical Neuroscience Center, Seoul National University Bundang Hospital, Seongnam, Republic of Korea; ^6^Department of Neurology, Seoul National University College of Medicine, Seoul, Republic of Korea

**Keywords:** anterior inferior cerebellar artery, vertigo, labyrinthitis, infarction, head-impulse test, sudden sensorineural hearing loss

## Abstract

**Introduction:**

Video head-impulse tests (video-HITs) often fail to detect anterior inferior cerebellar artery (AICA) infarction due to peripheral and central vestibular system involvement. Anecdotal studies suggest that video-HITs may reveal bilateral impairment in AICA infarction. However, the diagnostic utility of video-HITs has not been established, particularly when compared to labyrinthitis, which accounts for the majority of acute audiovestibular syndrome (AAVS) cases.

**Methods:**

We reviewed the medical records of consecutive patients presenting with new-onset acute hearing loss and spontaneous vertigo (i.e., AAVS) between March 2018 and July 2023 at a tertiary hospital in South Korea. Video-HIT patterns were categorized as follows: (1) ipsilaterally positive, (2) contralaterally positive, (3) bilaterally normal, and (4) bilaterally positive.

**Results:**

Twenty-eight patients with AICA infarction (mean age ± standard deviation = 67 ± 15 years; 14 men) and 51 with labyrinthitis (63 ± 17 years, 26 men) were included in the analyses. Among the 28 patients with AICA infarction, 15 presented with AAVS in isolation, without other co-morbid neurologic deficits (15/28, 54%). The vestibulo-ocular reflex (VOR) gains of ipsilesional horizontal canals (HCs) ranged from 0.21 to 1.22 (median = 0.81, interquartile range [IQR] = 0.50–0.89). However, those for contralateral HC gain ranged from 0.57 to 1.19 (median = 0.89 [IQR = 0.73–0.97]). Collectively, HITs were bilaterally positive in 13 patients (including 12 patients with bilaterally positive HITs for the horizontal canal), normal in eight, ipsilesionally positive in six, and contralesionally positive in one patient with AICA infarction. The VOR gains were typically decreased ipsilaterally in 28 (28/51, 55%), normal in 17 (17/51, 33%), and decreased bilaterally in six patients with labyrinthitis (6/51, 12%). Logistic regression analysis revealed that bilaterally positive HITs (*p* = 0.004) and multiple vascular risk factors (*p* = 0.043) were more frequently associated with AICA infarction than labyrinthitis.

**Discussion:**

Among patients presenting with AAVS, bilaterally positive HITs can be indicative of AICA infarction in patients with multiple vascular risk factors.

## Introduction

Acute audiovestibular syndrome (AAVS) is characterized by acute dizziness and vertigo accompanied by tinnitus, ear fullness, or hearing loss lasting >24 h (1, 2). AAVS represents a clinical emergency, necessitating a prompt and effective triage owing to its abrupt onset and possible sequelae of hearing loss. Although this condition can be attributed to a benign inner ear disorder (such as labyrinthitis), it may also arise from posterior circulation stroke (PCS) involving the territory of the anterior inferior cerebellar artery (AICA) (3, 4). Early recognition and intervention are crucial to minimize neurological sequelae and preserve auditory function (2, 5).

Anecdotal studies have investigated the diagnostic utility of vestibular or audiometric findings in the initial assessment of patients with AAVS. For instance, gaze-evoked nystagmus (GEN) and spontaneous nystagmus (SN) beating towards the ear with hearing loss usually indicate AICA infarction (6). Moreover, AICA infarction can be inferred from head-shaking nystagmus (HSN) beating contralateral to the direction of SN (6). However, conflicting results have been reported, which may be a reason for the low diagnostic yield of neurotologic tests for differentiating the two conditions (7).

The simple 3-step bedside oculomotor examination battery, Head-Impulse—Nystagmus—Test-of-Skew (HINTS), is efficient for evaluating patients with acute vestibular syndrome (8). Nevertheless, this test often fails in AICA infarction, as GEN may be absent in approximately 50% of patients, and positive HITs can occur due to inner ear damage (6, 9, 10). HINTS derivative, HINTS-plus (HINTS + finger rub test) (11), can be helpful in this instance. Patients with AAVS can be categorized as a central lesion under this triage strategy. Given that the condition of most patients with AAVS results from benign inner ear disorders (7, 12), neurotologic findings can help triage patients if properly cultivated (9, 13).

The results of HIT have been debated in AICA infarctions. A high prevalence of negative HITs (up to 50%) has been reported with AICA infarction (6, 7, 14). Contrastingly, positive HITs were observed with AICA infarction, potentially leading to misdiagnosis as peripheral vestibulopathy (9). Additionally, positive HITs may appear symmetrically on both sides, further complicating the diagnosis in a few patients (10). Given the ongoing discussion surrounding HIT results in patients with AAVS and the potential for misdiagnosis, we conducted a systematic investigation to determine the diagnostic utility of HITs in patients presenting with AAVS.

## Materials and methods

### Patients

The medical records of 160 consecutive patients with first-onset AAVS between March 2018 and July 2023 at the Korea University Medical Center were retrospectively reviewed by two authors (K.-T. K and B.-H. C). Data from 144 patients who underwent evaluation within 2 weeks of symptom onset were collected. Initially, we excluded 52 patients with no quantitative measurement of the VOR using video-HITs (*n* = 24), patients with no SN (*n* = 21), patients who exhibited ophthalmoplegia (*n* = 4), and patients who had PCS involving both sides of the brainstem or cerebellum (*n* = 3). We further excluded nine patients with a history of vestibulopathy that may have influenced the results of HITs: three with vestibular migraine, two with Meniere’s disease, two with multiple systemic atrophy, and two with a history of vestibular neuritis. We also excluded patients with improper HIT techniques, such as large head bounces with oppositely directed peak head velocity exceeding 50°/s (*n* = 2) or eye trace loss (*n* = 2) (15, 16).

The diagnosis of labyrinthitis was established when patients met three criteria: any evidence of acute vestibular impairment, determined as (1) presence of SN, (2) unilaterally or bilaterally positive video-HITs for HC or canal paresis >25% on the side of the new-onset hearing impairment, and (3) newly documented unilateral hearing impairment on audiometry. Accordingly, the pattern of SN and results of video-HITs may deviate from those observed in typical acute unilateral peripheral vestibulopathy. A subgroup analysis was conducted among patients meeting the criteria for sudden sensorineural hearing loss (SSNHL), defined by hearing loss of ≥30 dB over at least three contiguous audiometric frequencies within 72 h (17).

### Video-HITs

Head and eye movements were recorded using video-HITs (SLVNG; SLMED, Seoul, South Korea). The detailed methods for HITs have been described previously (18). The peak head acceleration exceeded 3,000 ° /s^2^ for the horizontal canals (HCs) and 2,000 ° /s^2^ for the vertical canals. VOR gains for each canal were measured for individual impulse trials as the ratio of the mean velocity of the eye divided by that of the head during the 40-ms time window centered at peak head acceleration.

The ipsilateral and contralateral sides were determined according to auditory symptoms. HIT patterns were classified as follows: (1) ipsilaterally positive, (2) contralaterally positive, (3) bilaterally normal, and (4) bilaterally positive. The results of video-HITs were determined to be positive (abnormal) when they fell outside the normal range of VOR gain from 19 healthy participants (8 men, mean age ± standard deviation [SD] = 62 ± 6 years). The reference range was defined as the mean ± 2SD. Normal values for the horizontal canal = 0.86–1.20; normal gain for the anterior canal = 0.75–1.23; normal gain for the posterior canal = 0.73–1.32 (19). Gain asymmetry between the HCs was defined as the difference in the VOR gains of the HCs on both sides (gain asymmetry = VOR gain_contralateral_ − VOR gain_ipsilateral_, with a negative value indicating a greater VOR gain on the side of auditory symptoms).

The VOR waveforms and corrective saccade patterns were analyzed separately from the gain calculation. The cumulative saccadic amplitude was averaged among the trials. A negative cumulative saccadic amplitude indicated corrective saccades in the direction of head rotation. Saccadic amplitude asymmetry was calculated from the sum of cumulative saccadic amplitudes between the sides using a formula (saccadic amplitude asymmetry = cumulative saccadic amplitude_ipsilateral_ – cumulative saccadic amplitude_contralateral_) / (cumulative saccadic amplitude_ipsilateral_ + cumulative saccadic amplitude_contralateral_; a negative value indicated a greater cumulative saccadic amplitude in the opposite ear of hearing impairment) (18). Wrong-way saccades were considered present when saccades occurred in the direction of head rotation, with peak eye velocity exceeding 60 ° /s, with the cutoff value determined by the main saccade sequence (20).

### Other neurotologic evaluations

The patients underwent bithermal caloric tests, as well as subjective visual vertical (SVV) and cervical and ocular vestibular-evoked myogenic potential (VEMPs) measurements.

Bithermal caloric tests were performed by alternately irrigating the ears for 30 s with cold and hot air (26° C and 50° C, respectively), and the asymmetry of the caloric responses was calculated using Jongkees’ formula. SVV was measured using a head-mounted device (NDI-150, M2S, Seoul, South Korea). Cervical and ocular VEMPs were recorded using a Nicolet Viking Select unit (Nicolet Biomedical, Madison, WI, United States). Cervical VEMPs were recorded while applying a short burst of alternating tones at 2.1 Hz monaurally via headphones. oVEMPs were elicited by tapping the hairline at the AFz using an electric reflex hammer (VIASYS Healthcare, CA, United States). Detailed descriptions of the reference ranges for each test have been described previously (21). All patients underwent pure tone audiometry using air- and bone-conducted signals in an acoustic booth. The hearing threshold was measured at 0.25, 0.5, 1 k, 2 k, 3 k, 4 k, and 8 k Hz. Pure tone asymmetry was calculated as between the sides using a formula (pure tone asymmetry = pure tone average_ipsilateral_ − pure tone average_contralateral_) / (pure tone average_ipsilateral_ + pure tone average_contralateral_).

### Brain and inner ear MRI

The MRI protocol included T1-, T2-, diffusion-weighted, gradient-echo axial, and T1-weighted sagittal images obtained with a 3.0-T unit, as previously described (22). For patients with labyrinthitis, dedicated inner ear MRIs were conducted using 3 T-MRI scanners (Magnetum Skyra, Magnetum Prisma, and Magnetum Vida units, Siemens, Erlangen, Germany) (22, 23). Positivity was determined by comparing the smothered polygonal region of interest of the enhancing lesion with that of the medulla.

### Statistical analysis

Continuous variables were compared using the Mann–Whitney U test or Student’s *t*-test, whereas nominal variables were compared using the χ ^2^ or Fisher’s exact test. In the regression analysis, all variables with *p* < 0.2 in the age- and sex-matched univariate analysis were included in the multivariable analysis. Variables with *p* < 0.05 in the multivariable analysis were considered significant.

Statistical analyses were performed using R (version 3.4.0; The R Foundation for Statistical Computing; Vienna, Austria; http://www.r-project.org).

## Results

### Clinical characteristics

We included 28 patients with AICA infarction (mean age ± SD = 67 ± 15 years, 14 men) and 51 with labyrinthitis (63 ± 17 years, 26 men) in the analyses. Detailed clinical profiles of the patients are presented in Table 1. In most patients, the lesion was confined to the territory of the AICA; however, five patients had lesions in the territories of posterior (*n* = 3) and middle (*n* = 2) cerebral arteries. Patients with AICA infarction predominantly presented with acute dizziness/vertigo and hearing impairment (i.e., AAVS). However, they exhibited other focal neurologic deficits, including appendicular ataxia (*n* = 9), dysarthria (*n* = 8), diplopia (*n* = 6), facial palsy (*n* = 5), sensory changes (*n* = 4), motor weakness (*n* = 4), visual field defects (*n* = 4), and dysphagia (*n* = 1). Fifteen patients presented with isolated AAVS (15/28; 54%; Supplementary Table S1). Initial MRI findings were false-negative in four patients (4/28, 14%). However, they became positive on follow-up MRIs. Tinnitus (32/51 [63%] vs. 11/28 [39%], *p* = 0.045) and ear fullness (22/51 [43%] vs. 5/28 [18%], *p* = 0.027) were less common in patients with AICA infarction compared to patients with labyrinthitis (Table 1). Labyrinthitis was radiologically documented on inner ear MRI in 19 patients (19/48, 40%; excluding three with incomplete evaluation of MRI).

**Table 1:** Clinical characteristics of the patients.

	AICA infarction (*n* = 28)	Labyrinthitis (*n* = 51)	*p* value
Sex, men (%)	15 (54)	26 (51)	0.825
Age, mean ± SD, years	66 ± 13	60 ± 18	0.113
Lesion side, right (%)	13 (46)	25 (49)	≈ 1.000
Tinnitus (%)	11 (39)	32 (63)	**0.045**
Ear fullness (%)	5 (18)	22 (43)	**0.027**
Hearing loss (%)	23 (82)	46 (90)	0.314
Diabetes mellitus (%)	10 (36)	13 (26)	0.339
Hypertension (%)	21 (75)	28 (55)	0.078
Dyslipidemia (%)	9 (32)	5 (10)	**0.013**
Smoking (%)	5 (18)	2 (4)	0.090
Prior stroke/TIA (%)	5 (18)	2 (4)	0.090
CAOD/myocardial infarction (%)	2 (7)	3 (6)	≈ 1.000
Atrial fibrillation (%)	1 (4)	1 (2)	≈ 1.000
Vascular risk factors (%)*	2 (1–3)	1 (0–2)	**0.006**
Initial false negative MRI for stroke (%)	4 (14)	–	
MRI-positive labyrinthitis (%)^a^	–	19 (40)	

AICA, anterior inferior cerebellar artery; CAOD, coronary artery occlusive disease; SD, standard deviation; TIA, transient ischemic attack.

*Counts of vascular risk factors that include diabetes mellitus, hypertension, dyslipidemia, smoking, prior stroke/TIA, coronary artery occlusive disease/myocardial infarction, and atrial fibrillation.

a Excluding three patients without incomplete evaluation of MRI. Bold values indicate statistical significance.

### Head-impulse gain of the VOR

The HIT results are summarized in Table 2. In patients with AICA infarction, the SN direction was mostly contralesional (16/26, 62%, excluding two with pure downbeat nystagmus). SN was observed in various patterns: purely horizontal (*n* = 11), horizontal-vertical (*n* = 9), horizontal-vertical-torsional (*n* = 5), purely vertical (*n* = 2), and horizontal-torsional (*n* = 1). The horizontal slow-phase velocity (SPV) of SN ranged from −4.0 to 10.9 ° /s in patients with AICA infarction (median = 0 ° /s [IQR = −1.5–1.7], with a negative value indicating SN beating towards the lesioned ear).

**Table 2:** Results of HITs and other neurotological findings in patients with AICA infarction and labyrinthitis.

	AICA infarction (*n* = 28)	Labyrinthitis (*n* = 51)	*p* value
**Onset-to-evaluation, median, days (IQR)**	7 (5–10)	4 (2–7)	**0.003**
**Ipsilesional SN (%)^a^**	12 (46)	14 (28)	0.114
**Horizontal SPV of SN, median (IQR), °/s^b^**	0 (−1.5–1.7)	2.0 (−0.5–3.4)	**0.043**
**HITs**			
Involved side (%)			**0.011**
*Normal*	8 (29)	17 (33)	0.663
*Unilaterally positive (I)*	6 (21)	28 (55)	**0.004**
*Unilaterally positive (C)*	1 (4)	0 (0)	0.354
*Bilaterally positive*	13 (46)	6 (12)	**0.001**
Involved canal (%)			
*HC, (I)*	18 (64)	33 (65)	0.970
*AC, (I)*	11 (39)	7 (14)	**0.010**
*PC, (I)*	14 (50)	10 (20)	**0.005**
*HC, (C)*	13 (46)	6 (12)	**0.001**
*AC, (C)*	8 (29)	0 (0)	**< 0.001**
*PC, (C)*	12 (43)	2 (4)	**< 0.001**
**Wrong-way saccades (%)**	6 (21)	14 (28)	0.556
**Gain asymmetry, median (IQR), %**	0.10 (0.02–0.34)	0.22 (0.10–0.40)	0.134
**Negative gain asymmetry (%)**	6 (21)	8 (16)	0.523
**Canal asymmetry, median (IQR), %^c^**	29 (−4–51)	36 (2–74)	0.786
**Canal paresis (%)**	14/24 (58)	31/50 (62)	0.762
**Pure tone asymmetry, median (IQR)**	22 (10–57)	52 (27–67)	0.068
**Pure tone average, ipsilesional ear, median (IQR), dB**	60 (39–92)	58 (36–110)	0.991
**SSNHL (%)**	14 (50)	37 (73)	**0.045**
**Central HINTS (%)**	15 (58)	18 (35)	0.060
*Skew deviation*	5 (18)	0 (0)	**0.004**
*Horizontal gaze-evoked nystagmus*	9 (32)	1 (2)	**<0.001**
*Normal HITs*	8 (29)	17 (33)	0.663
**Head-shaking nystagmus (Normal/I/C/Perverted)**	8/6/11/3	17/4/28/2	0.188
**oVEMP (Normal/I/C/B)**	12/10/1/1	7/13/1/1	0.651
**cVEMP (Normal/I/C/B)**	4/10/2/4	12/26/2/2	0.235
**SVV (Normal/I/C)**	4/11/4	4/11/2	0.753

a Except two patients with pure downbeat nystagmus.

b Negative value indicates the direction of nystagmus is towards the ear with hearing impairment.

c Negative value indicates the canal asymmetry is towards the ear with hearing impairment.

AICA, anterior inferior cerebellar artery; C, contralesional; cVEMP, cervical vestibular-evoked myogenic potential; HIT, head-impulse test; I, ipsilesional; IQR, interquartile range; oVEMP, ocular VEMP; SN, spontaneous nystagmus without visual fixation; SPV, slow-phase velocity; SSNHL, sudden sensorineural hearing loss. Bold values indicate statistical significance.

Conversely, the SN direction was contralesional in 37 patients and ipsilesional in 14 patients with labyrinthitis. SN directions were predominantly horizontal-torsional (*n* = 27), followed by horizontal-vertical with (*n* = 12) or without a torsional component (*n* = 8), pure horizontal (*n* = 3), and vertical-torsional (*n* = 1). The SPV of SN ranged from −19.0 to 10.1 ° /s (median = 2.0 [IQR = –0.5–3.4]).

**Figure 1:**
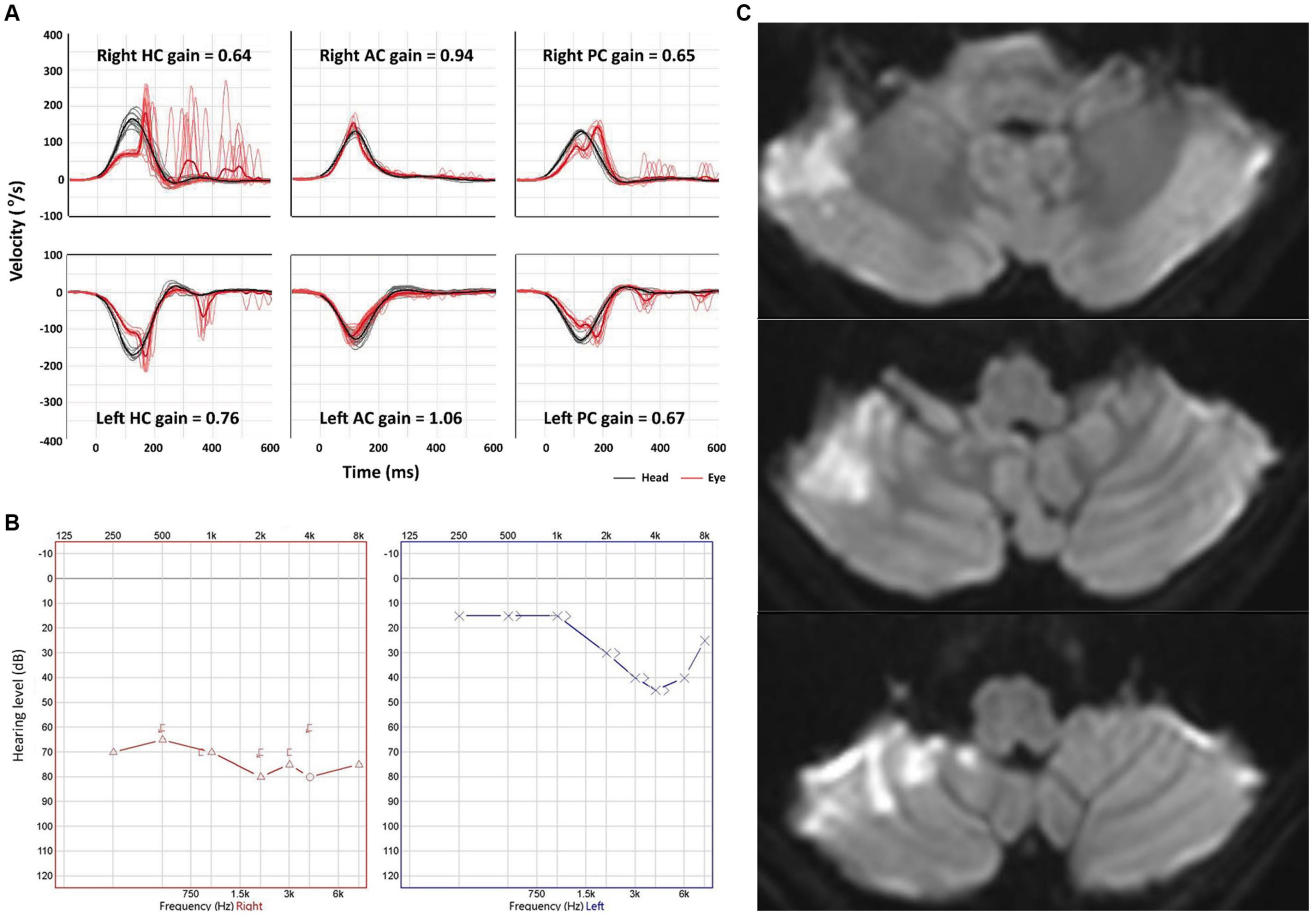
Neurotologic and imaging findings in a representative patient with anterior inferior cerebellar artery (AICA) infarction. **(A)** Video head-impulse tests reveal a decreased gain of vestibulo-ocular reflex for both HCs and PCs bilaterally. **(B)** Pure tone audiometry shows a sensorineural hearing loss at 73 dB in the right ear. **(C)** Diffuse weighted images show an acute infarction in the right AICA territory. AC, anterior canal; HC, horizontal canal; PC, posterior canal.

**Figure 2:**
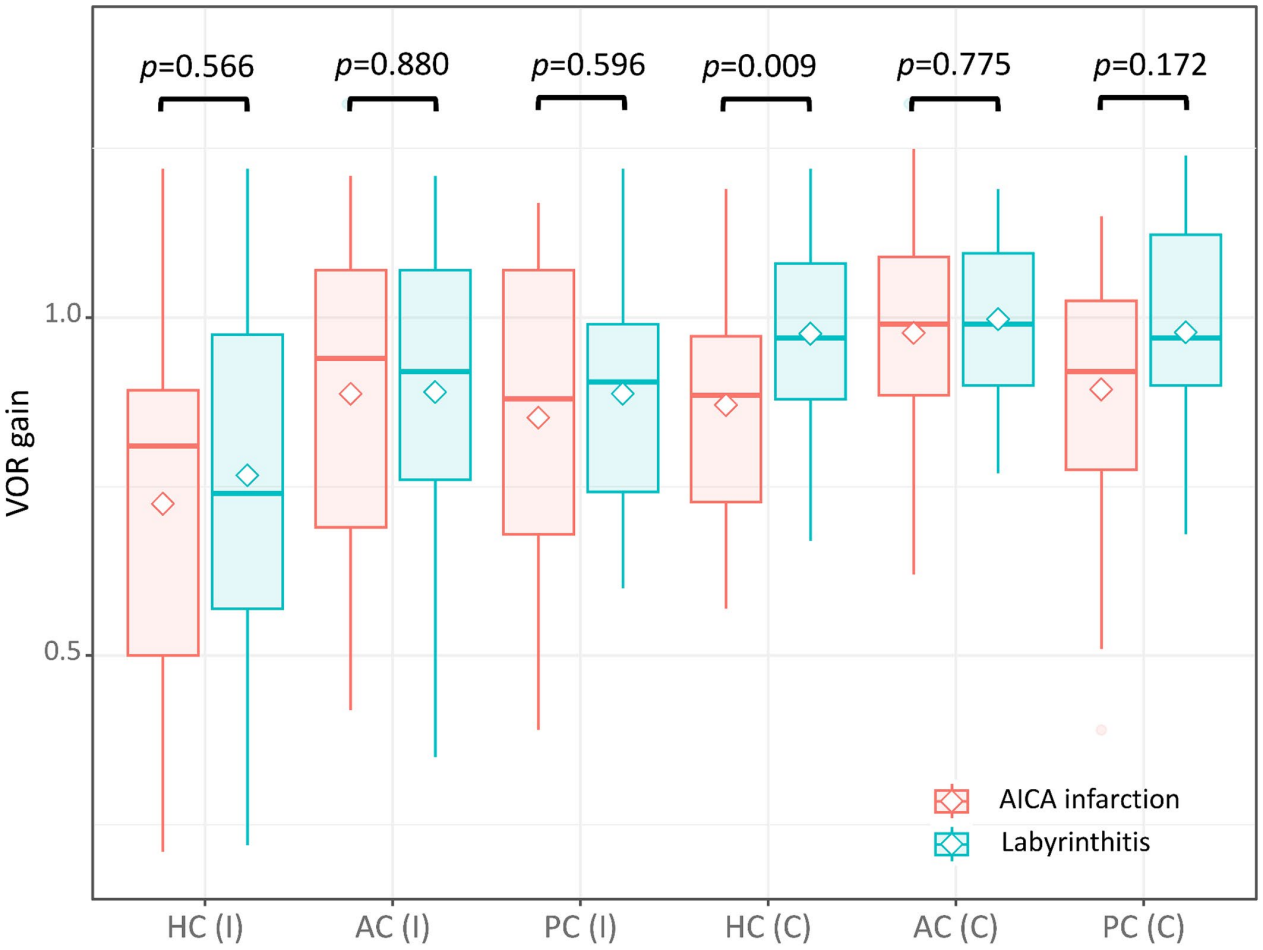
The VOR gain of each semicircular canal in patients with AICA and labyrinthitis. The VOR gain was decreased for the contralesional HC in patients with AICA infarction compared to those with labyrinthitis (median = 0.89 [interquartile range = 0.73–0.97] vs. 0.97 [0.88–1.08], *p* = 0.009). Otherwise, the gain did not differ between the two for other semicircular canals (0.81 [0.50–0.89] vs. 0.74 [0.57–0.98] for ipsilesional HC; 0.94 [0.69–1.07] vs. 0.92 [0.76–1.07] for ipsilesional AC; 0.88 [0.68–1.07] vs. 0.91 [0.74–0.99] for ipsilesional PC; 0.99 [0.89–1.09] vs. 0.99 [0.9–1.10] for contralesional AC; 0.92 [0.78–1.03] vs. 0.97 [0.90–1.12] for contralesional PC). AC, anterior canal; C, contralateral; HC, horizontal canal; I, ipsilateral; PC, posterior canal; VOR, vestibulo-ocular reflex.

The VOR gains of ipsilesional HCs ranged from 0.21 to 1.22 (median = 0.81 [IQR = 0.50–0.89]) in patients with AICA infarction. Among these patients, video-HITs were bilaterally positive in 13 patients (including 12 patients with bilaterally positive video-HITs for the HC), normal in eight, ipsilesionally positive in six, and contralesionally positive in one patient (Figure 1; Table 2). The contralateral HC gain ranged from 0.57 to 1.19 (median = 0.89 [IQR = 0.73–0.97]), and the gain asymmetry ranged from −0.32 to 0.57 (0.10 [0.02–0.34]). Six patients (6/28, 21%) with AICA infarction showed negative gain asymmetry. The VOR gain was decreased for contralesional HC in patients with AICA infarction compared to those with labyrinthitis (median = 0.87 [IQR = 0.73–0.97] vs. 0.97 [0.88–1.11], *p* = 0.009). Otherwise, the gain did not differ for other semicircular canals (Figure 2).

Seventeen patients (17/51, 33%) with labyrinthitis demonstrated normal VOR gains in all canals. In these patients, the vestibular deficit was determined based on canal paresis of >25% observed during bithermal caloric tests. The VOR gains of ipsilesional HCs ranged from 0.22 to 1.22 (median = 0.74 [IQR = 0.57–0. 89]) in patients with labyrinthitis. The contralateral HC gain ranged from 0.67 to 1.24 (median = 0.97 [IQR = 0.88–1.08]). The gain asymmetry ranged from −0.21 to 0.62 (median = 0.22 [0.10–0.40]), and eight patients (8/51, 16%) showed a negative gain asymmetry. Collectively, the VOR gains were typically decreased ipsilaterally in 28 (28/51, 55%), normal in 17 (17/51, 33%; Figure 2), and decreased bilaterally in six patients with labyrinthitis (6/51, 12%; all patients showed bilateral positive video-HITs for the HC). Furthermore, the gain was abnormal in ipsilesional PC (*n* = 10), AC (*n* = 7), and contralesional PC (*n* = 2; Table 2).

Among the 15 patients with AICA infarction with AAVS in isolation, SN was purely horizontal in five patients, horizontal-vertical with or without a torsional component in four patients, and purely vertical in the remaining two patients.

### Audiometry

In the lesioned ears, the pure tone average did not differ between AICA infarction and labyrinthitis (median [IQR] = 60 dB [39–62] vs. 58 dB [36–110], *p* = 0.991). Additionally, pure tone asymmetry did not differ between the two groups (22% [10–57] vs. 52% [27–67], *p* = 0.068). The number of patients fulfilling the SSNHL criteria was lower in those with AICA infarction than in those with labyrinthitis (14/28 [50%] vs. 37/51 [73%], *p* = 0.045).

### Other neurotologic evaluations

Patients with AICA infarction exhibited additional central neurotological findings, including horizontal GEN (*n* = 9), skew deviation (*n* = 5), saccadic hypermetria (*n* = 3), and central positional nystagmus (*n* = 2). HSN was observed in 20 (20/28, 71%) patients with AICA infarction, predominantly in a horizontal direction in 17 (contralesional in 11 and ipsilesional in six) and perverted downbeat in the remaining three. Canal paresis was observed in 14 patients with AICA infarction (14/24, 58%). The oVEMP was normal in 12 patients, whereas 12 patients showed abnormal responses during stimulation of the ipsilesional ear (*n* = 10), contralesional ear (*n* = 1), or both (*n* = 1). cVEMP was normal in four patients, whereas 16 patients showed abnormal responses either during stimulation of the ipsilesional ear (*n* = 10), contralesional ear (*n* = 2), or both (*n* = 4). The SVV was tilted to either the lesioned side (*n* = 11), healthy side (*n* = 4), or normal (*n* = 4; Table 2).

In contrast, horizontal GEN was observed in one patient with labyrinthitis (1/51, 2%). HSN was observed in 34 patients with labyrinthitis (34/51, 67%), most of whom were beating contralesionally (28/34, 82%). Two patients exhibited perverted HSN (2/51, 4%; Table 2). Central HINTS was similarly observed in patients with AICA infarction and labyrinthitis (15/28 [58] vs. 18/51 [35%], *p* = 0.060). Canal paresis was observed in 31 patients with labyrinthitis (31/50, 62%). The oVEMP was normal in seven patients, whereas 15 patients showed abnormal responses either during stimulation of the ipsilesional ear (*n* = 13), contralesional ear (*n* = 1), or both (*n* = 1). The cVEMP was normal in 12 patients; however, 30 patients showed abnormal responses during stimulation of the ipsilesional ear (*n* = 26), contralesional ear (*n* = 2), or both (*n* = 2). The SVV was tilted to either the lesioned side (*n* = 11), healthy side (*n* = 2), or normal (*n* = 4; Table 2).

### Characteristics of video-HITs of patients with AICA infarction versus labyrinthitis

Bilaterally positive video-HITs were more frequently observed in patients with AICA infarction than in those with labyrinthitis (13/28 [46%] vs. 6/51 [12%]; *p* = 0.001). Wrong-way saccades showed no significant difference between the two groups (6/28 [21%] vs. 14/51[28%]; *p* = 0.556). Similarly, a negative gain asymmetry was observed in both groups (6/28 [21%] vs. 8/51 [16%]; *p* = 0.523; Table 2).

Logistic regression analysis revealed that bilaterally positive video-HITs and multiple vascular risk factors were frequently associated with AICA infarction (Table 3).

**Table 3:** Prediction of AICA infarction compared to labyrinthitis.

	Unadjusted OR (95% CI)	Age and Sex adjusted OR (95% CI)	Multivariable analysis (95% CI)†	*p* value for †
Age	1.03 (0.99–1.06)	–	1.00 (0.96–1.03)	0.801
Male sex	0.90 (0.36–2.27)	–	1.28 (0.42–3.90)	0.665
Vascular risk factors^a^	1.78 (1.17–2.68)	1.69 (1.09–2.60)	1.65 (1.02–2.69)	**0.043**
Bilaterally positive HITs	7.50 (2.43–23.19)	6.77 (2.10–21.85)	6.12 (1.77–21.14)	**0.004**
Wrong-way saccades during HITs	0.72 (0.24–2.15)	0.79 (0.26–2.42)	–	
VOR gain, HC, ipsilesional	0.54 (0.09–3.18)	0.51 (0.09–3.10)	–	
Pure tone asymmetry	0.14 (0.02–0.81)	0.15 (0.02–1.01)	0.14 (0.02–1.17)	0.070

The lesioned side was assigned according to the hearing impairment.

a Counts of vascular risk factors that include diabetes mellitus, hypertension, dyslipidemia, smoking, prior stroke/TIA, coronary artery occlusive disease/myocardial infarction, and atrial fibrillation.

AICA, anterior inferior cerebellar artery; CI, confidence interval; HC, horizontal canal; HITs, head-impulse tests; OR, odds ratio; VOR, vestibulo-ocular reflex. Bold values indicate statistical significance. ^†^Indicate that *p*-values for multivariable analysis is presented.

### Subgroup analysis

1. ***Patients presenting with AAVS in isolation (without any other neurologic deficit other than neurotologic findings)***.
  Among patients presenting with AAVS in isolation (15/28, 54%), logistic regression analysis revealed that bilaterally positive video-HITs (odds ratio, 95% confidence interval [CI] = 6.45 [1.50–27.77], *p* = 0.012) were associated with AICA infarction. Conversely, large pure tone asymmetry was associated with labyrinthitis (0.05 [0.004–0.73], *p* = 0.039; Table 4).
2. ***Patients with hearing impairment fulfilling SSNHL***.
  In patients with SSNHL, logistic regression analysis showed that bilaterally positive video-HITs were associated with AICA infarction (OR [95% CI] = 9.85 [2.00–48.43], *p* = 0.005; Supplementary Table S1).

**Table 4:** Sensitivity analysis of prediction of AICA infarction compared to labyrinthitis among patients presenting with AAVS in isolation.

	Unadjusted OR (95% CI)	Age and Sex adjusted OR (95% CI)	Multivariable analysis (95% CI) †	*p* value for †
Age	1.04 (0.99–1.09)	–	1.02 (0.97–1.07)	0.490
Male sex	0.75 (0.23–2.44)	–	1.05 (0.26–4.25)	0.944
Vascular risk factors	1.57 (0.92–2.68)	1.33 (0.73–2.41)	–	
Bilaterally positive HITs	8.19 (2.18–30.85)	6.62 (1.64–26.78)	6.45 (1.50–27.77)	**0.012**
Wrong-way saccades during HITs	0.69 (0.17–2.85)	0.72 (0.17–3.06)	–	
VOR gain, HC, ipsilesional	0.55 (0.06–5.54)	0.58 (0.06–5.89)	–	
Pure tone asymmetry	0.05 (0.01–0.59)	0.05 (0.004–0.73)	0.05 (0.003–0.86)	**0.039**

AAVS, acute audiovestibular syndrome; AICA, anterior inferior cerebellar artery; CI, confidence interval; HC, horizontal canal; HITs, head-impulse tests; OR, odds ratio; VOR, vestibulo-ocular reflex. The lesioned side was assigned according to the hearing impairment. Bold values indicate statistical significance. ^†^Indicate that *p*-values for multivariable analysis is presented.

## Discussion

The main findings of our study can be summarized as follows: (1) Video-HITs were frequently bilaterally positive for each canal in patients with AICA infarction compared to those in patients with labyrinthitis; (2) along with the presence of vascular risk factors, bilaterally positive video-HITs are also associated with AICA infarction; and (3) this trend was replicated in subgroup analyses among patients fulfilling SSNHL and those presenting with AAVS in isolation.

Video-HITs offer valuable insights into the integrity of the VOR in patients presenting with AVS (24). Patients with VN typically exhibit a largely decreased gain of the VOR followed by covert or overt saccades for the involved canals. Conversely, patients with PCS can present with either normal (25, 26) or abnormal responses (18, 27), depending on the central VOR pathway involvement (10, 28). Conventionally, a normal VOR gain considerably suggests a central lesion in patients presenting with AVS (26, 29). However, video-HITs often prove unreliable in AICA infarction (9) owing to the involvement of the internal auditory artery, a branch of the AICA, which supplies the inner ear (30, 31). The AICA also supplies the lateral pons, middle cerebellar peduncle, and anterior inferior cerebellum, including the flocculus (32). Consequently, AICA infarction results in facial palsy, motor weakness, sensory deficit, ataxia (4), and features of combined central and peripheral vestibulopathy owing to the inner ear and flocculus involvement (4, 9, 14).

Given the limitations of video-HITs in AICA infarction (9), exploring alternative algorithms for triaging patients with AAVS is imperative (4, 7, 33). New-onset hearing impairment with multiple vascular risk factors is considered a central cause when associated with acute vestibular impairment (HINTS-plus) (11, 34). However, this approach cannot differentiate labyrinthitis, which commonly causes AAVS (7, 9, 35). Our study suggests that although regarded as a sign indicating a peripheral vestibulopathy, positive video-HITs can infer a vascular etiology when impaired bilaterally, suggesting an involvement of the flocculus or medial vestibular nucleus in patients presenting with AAVS (11).

### Bilaterally positive HITs in AICA infarction

Video-HITs reveal a decrease in VOR gain in approximately 30% of PCS (29), particularly when the direct VOR pathway is affected (27, 28). In such cases, patients often exhibit bilaterally positive video-HITs (10, 14, 18) because central neural substrates, such as the medial vestibular nucleus (27), nucleus prepositus hypoglossi (NPH) (36), or flocculus (37), participate in the direct VOR system on both sides. The magnetic search coil also supports these findings by demonstrating reduced contralesional VOR gains in the PCS (10, 38). Furthermore, bilaterally positive video-HITs remain valid for differentiation in subsets of patients with isolated AAVS or those with SSNHL. Our observations indicate that the clinical approach should be stratified differently when auditory symptoms are present. Additionally, results of video-HITs should be appreciated contralateral to the side of auditory symptoms to distinguish a debilitating AICA infarction from a benign inflammatory disorder involving the inner ear.

### Contralesional decrement of VOR gain in peripheral vestibulopathy

The VOR gain can also decrease contralesionally in peripheral vestibulopathy (10, 39). However, the decrement is usually not as profound as those from the ipsilesional side, ranging from 0.51 to 0.76 (10, 39). Consequently, bilaterally positive responses can be observed in peripheral vestibulopathy, although in a few patients with VN (~9%) (18). Similarly, the VOR gains are decreased bilaterally in 12% of our patients with labyrinthitis.

The decrease of VOR gain on the healthy side may be attributed to adaptation following damage in the labyrinth or primary vestibular afferents (40). This central adaptation is mediated by modulating the neural discharges in the vestibular nuclei: when the neuronal activity in the vestibular nuclei is measured in a cat or guinea pig, the resting rate slightly increases, whereas the rotation sensitivity of type I vestibular neurons decreases immediately after contralateral labyrinthectomy (less robust than the ipsilateral neuron) (41–43). This neuronal adaptation consequently causes the decrement of VOR gain in the contralateral side of labyrinthectomy. Nevertheless, clinical studies indicate that the adaptational VOR decrement on the unaffected side is generally less pronounced than the significant reduction observed in PCS, not reaching the threshold considered abnormal (14, 27, 28, 37).

### Ipsilesional SN or negative gain asymmetry in AAVS

Negative gain asymmetry, indicating ipsilateral gain bias, was present in 21% of patients with AICA infarction. Similarly, ipsilesional SN was present in 46% of AICA infarctions. Ipsilesional SN has been reported in AICA, posterior inferior cerebellar artery (PICA), and superior cerebellar artery infarctions (4, 6, 44, 45). The flocculus sends inhibitory projection onto the ipsilateral vestibular nucleus (46–48). Vestibular bias can be reversed by denervation of the GABAergic cerebellar efferent pathway, predominantly innervating the ipsilateral vestibular nuclei complex (47, 49). Thus, unilateral lesions of flocculus lead to ipsilesional nystagmus in animals and humans (37, 50).

However, ipsilesional SN (nystagmus beating towards the hearing impairment) and negative gain asymmetry were also observed in 28% of our patients with labyrinthitis. This phenomenon can be explained by recovery nystagmus attributed to the recovery from vestibular deficit (51). It may have been frequently observed because the time of evaluation from onset ranged up to 14 days in our patients. The diagnoses slightly differ across the literature, and the pattern of SN remains elusive in labyrinthitis. Thus, the proportion of ipsilesional nystagmus and bilaterally positive HITs may differ depending on eligibility and patient characteristics. Our findings suggest that nystagmus beating towards the side of hearing impairment does not necessarily indicate a central lesion in AAVS. Therefore, rather than focusing solely on the SN direction, considering video-HITs in the SN direction is also essential, which is an aspect that has previously been overlooked in PCS (18).

### Diagnosis of labyrinthitis

The results of video-HITs and caloric tests in labyrinthitis can deviate from those typical acute unilateral peripheral vestibulopathy in our study. This discrepancy may be due to the lenient inclusion criteria for labyrinthitis. We adopted broad inclusion criteria because the pattern of vestibular involvement in patients with labyrinthitis requires further elucidation (52). Given the MRI findings also differ, the underlying etiology of labyrinthitis may differ from those of vestibular neuritis (23, 53–57). Indeed, canal involvement based on video-HITs is distinct from that seen in typical acute unilateral peripheral vestibulopathy (52, 58–61). As stated, the clinical significance of ipsilesional SN also remains to be delineated in AAVS. Thus, strict eligibility adhering to the Barany Society’s definition could provide additional implications for future studies in patients presenting with AAVS.

### Normal video-HITs in labyrinthitis

One-third of our patients with labyrinthitis exhibited normal video-HIT results based on VOR gain measurements. Vestibular impairment may only become evident during low-frequency stimulation in those patients. Likewise, abnormal caloric responses in the presence of preserved video-HITs (caloric-HIT dissociation) were observed in approximately 7% of patients with VN (10, 52, 62). This observation can be explained by marginal vestibular deficits in VN, resulting in marginal decrements of VOR gain (14). Alternatively, vestibular impairment can evolve during the acute phase in VN (63). Most of all, simple dichotomization into normal versus abnormal based on VOR gain can have limited diagnostic yield for estimating the vestibular deficit (10, 18, 64, 65). In this context, caloric tests and incorporating corrective saccades may complement the diagnosis of peripheral vestibulopathy (10, 18, 64, 66, 67).

### False-negative MRIs in AICA infarction

Initial MRIs were false-negative in 14% of AICA infarctions. This mirrors the pattern observed in PICA stroke, where MRIs often fail to provide conclusive evidence, and the diagnosis predominantly relies on central neurotologic findings (8, 26, 27). In such cases, differentiation can be challenging, particularly when patients present with AVS or AAVS in isolation (68). Our observations suggest that meticulous analysis of video-HITs can improve the accuracy of neurotologic evaluations and help overcome the limitations of imaging studies in diagnosing AAVS.

### Caveats and limitations of our study and suggestions for future studies

Our study had some limitations. Some cases of labyrinthitis may have resulted from vascular etiology. Epidemiologic studies indicate that the risk of future stroke is higher in patients with labyrinthitis or SSNHL (69, 70). It implies the involvement of a vascular mechanism in the pathogenesis of SSNHL, at least in some older patients. Second, the eligibility of our study is rather broad to include those with symptom onset within 14 days. Thus, whether our findings are valid in patients with AAVS with hyperacute stages (<72 h) is unknown. Third, the determination of positive responses was based on simple VOR gain measurement, which may not be optimal for analyzing the results of video-HITs. Thus, a future study using corrective saccades in a larger number of patients may clarify this.

In conclusion, bilaterally positive video-HITs can differentiate AICA infarction from labyrinthitis. Furthermore, the results of video-HITs contralateral to the side of auditory symptoms should be considered in order to effectively distinguish debilitating AICA infarction from a benign inflammatory disorder involving the inner ear.

## Data Availability

Anonymized data will be made available upon reasonable request from any qualified investigator.
