# Supplementary material for: Bilaterally positive head-impulse tests can differentiate AICA infarction from labyrinthitis
Source: Front Neurol. 2024 Aug 29;15:1448989. doi: 10.3389/fneur.2024.1448989 (PMC11390645; doi:10.3389/fneur.2024.1448989)
Supplement: Supplementary file 1 [file Data_Sheet_1.docx]

**Supplementary Table 1. Neurotologic findings of the patients with AICA infarction presenting with AAVS in isolation**

| **Pt no.** | **Age/Sex** | **Video head-impulse test VOR gain** | | | | | | **Canal paresis (%)*** | **Pure tone asymmetry*** |
| --- | --- | --- | --- | --- | --- | --- | --- | --- | --- |
|  |  | **HC (I)** | **HC (C)** | **AC (I)** | **AC (C)** | **PC (I)** | **PC (C)** |  |  |
| **1** | 78/M | 0.64 | 0.77 | 1.15 | 1.25 | 0.63 | 0.72 | 72 | 0.27 |
| **2** | 84/F | 0.47 | 0.64 | 0.75 | 0.98 | 0.78 | 0.77 | Not done | 0.54 |
| **3** | 69/M | 1.16 | 1.18 | 1.19 | 1.23 | 0.72 | 0.71 | 26 | 0.21 |
| **4** | 77/F | 0.78 | 0.81 | 0.93 | 0.88 | 0.88 | 0.78 | -2 | 0.22 |
| **5** | 64/M | 0.82 | 0.85 | 0.71 | 0.94 | 0.79 | 0.79 | 28 | 0 |
| **6** | 65/F | 0.84 | 0.96 | 0.80 | 1.03 | 0.91 | 0.97 | -12 | 0.31 |
| **7** | 66/M | 0.50 | 1.07 | 1.04 | 1.10 | 1.11 | 1.15 | 41 | 0.49 |
| **8** | 60/M | 0.84 | 0.84 | 0.64 | 0.71 | 0.61 | 0.88 | Not done | 0.13 |
| **9** | 62/F | 1.03 | 0.99 | 0.99 | 0.95 | 1.15 | 1.13 | 30 | 0.07 |
| **10** | 54/M | 0.36 | 0.83 | 0.65 | 1.04 | 1.05 | 1.01 | Not done | 0.16 |
| **11** | 78/M | 0.83 | Not done | Not done | 0.73 | Not done | Not done | -6 | -0.02 |
| **12** | 77/M | 0.56 | 0.94 | 0.64 | 0.75 | 0.75 | 1.15 | 48 | 0.49 |
| **13** | 75/F | 0.88 | 0.98 | 1.10 | 1.15 | 0.97 | 0.95 | 1 | 0 |
| **14** | 74/M | 0.39 | 0.72 | 0.42 | 1.09 | 0.39 | 0.39 | 74 | 0.70 |
| **15** | 57/F | 0.73 | 0.73 | 0.68 | 0.79 | 0.71 | 0.71 | 57 | 0.22 |

AAVS = acute audiovestibular syndrome, AC = anterior canal, AICA = anterior inferior cerebellar artery, C = contralesional, HC = horizontal canal, HITs = head-impulse tests, I = ipsilesional, PC = posterior canal, VOR = vestibulo-ocular reflex

The lesioned side was assigned according to the hearing impairment.

*Negative indicates canal asymmetry or pure tone asymmetry toward the unaffected side

**Supplementary Table 2. Sensitivity analysis of prediction of AICA infarction compared to labyrinthitis among patients with hearing impairment fulfilling SSNHL**

|  | **Unadjusted OR (95% CI)** | **Age and Sex adjusted OR (95% CI)** | **Multivariate analysis (95% CI)*** | ***p* value for *** |
| --- | --- | --- | --- | --- |
| **Age** | 1.02 (0.98–1.07) | - | 1.00 (0.95–1.05) | 0.932 |
| **Male sex** | 0.87 (0.26–2.91) | - | 0.77 (0.20–3.10) | 0.714 |
| **Vascular risk factors** | 1.42 (0.86–2.34) | 1.31 (0.74–2.33) |  |  |
| **Bilaterally positive HITs** | 9.43 (2.21–40.24) | 9.85 (2.00–48.43) | 9.85 (2.00–48.43) | **0.005** |
| **Wrong-way saccades during HITs** | 0.68 (0.16–2.90) | 0.65 (0.15–2.85) |  |  |
| **VOR gain, HC, ipsilesional** | 0.24 (0.03–2.26) | 0.23 (0.02–2.18) |  |  |
| **Pure tone asymmetry** | 0.20 (0.01–3.92) | 0.34 (0.01–9.67) |  |  |

AAVS = acute audiovestibular syndrome, HC = horizontal canal, HITs = head-impulse tests, OR = odds ratio, VOR = vestibulo-ocular reflex

The lesioned side was assigned according to the hearing impairment.
